# 854. From Burn Unit to Home: Bridging the Gap with Nursing & Pharmacy Innovation

**DOI:** 10.1093/jbcr/irag033.324

**Published:** 2026-04-06

**Authors:** Janie Faris, Alina Ruiz, Spencer Simko, Holly Coco, Amber M Ewing, Rebecca Coffey

**Affiliations:** Parkland Health, Grapevine, TX; Parkland Health, Garland, TX; University of Texas Southwestern Medical Center, Dallas, TX; University of Texas Southwestern Medical Center, Dallas, TX; Parkland Health, Fate, TX; Parkland Health, Dallas, TX

## Abstract

**Introduction:**

In 2023 our burn center observed an increase in unplanned readmissions, secondary to issues with understanding wound care, availability of wound care supplies and pain control. Nursing and pharmacy collaborated to establish multiple initiatives to improve transitions of care from inpatient to outpatient. The primary objective of this study was to evaluate the effectiveness of nursing and pharmacy interventions to decrease unplanned readmissions.

**Methods:**

A retrospective chart review was conducted to compare patients admitted during the pre-intervention group Oct 1, 2022 - Oct 31, 2023, to the post group Nov 1, 2023 - Nov 30, 2024. Interventions included: multimodal nursing education to improve patient compliance with pain medication, development and implementation of educational wound care instructions both written and videos, and post discharge burn nurse and pharmacy follow up phone calls. Descriptive statistics were used to represent the data.

**Results:**

A total of 482 patients in the pre-intervention group and 838 patients in the post-intervention group were included. Despite the implementation of multiple interventions, the rates of readmissions were similar. The implementation of the interventions identified several key issues resulting in additional strategies. These issues included: difficulty identifying and tracking all burn patients, including those in observation status or ED thruput, availability of patients to access online education resources after discharge, limited access to telephone service which dramatically reduced follow up calls, and availability of discharge education in multiple languages. See table 1 and table 2.

**Conclusions:**

Implementing a nursing and pharmacy intervention is a complex undertaking requiring significant resources. Within one year, our departments have increased patient follow-up calls, patient education materials in various formats and languages, and access to supplies and ways to improve patient care. Ongoing work continues to address persistent challenges in readmission rates.

**Applicability of Research to Practice:**

decreasing overall hospital re-admission rates post burn injury is a probably for all burn units and results in significant health care costs to the patient and hospital systems.

**Funding for the study:**

N/A.

